# Excited-State Intramolecular Proton Transfer: A Short Introductory Review

**DOI:** 10.3390/molecules26051475

**Published:** 2021-03-09

**Authors:** Hem C. Joshi, Liudmil Antonov

**Affiliations:** 1 Institute for Plasma Research, Bhat, Gandhinagar 382428, India; hem_sup@yahoo.co.uk; 2 Institute of Electronics, Bulgarian Academy of Sciences, 1784 Sofia, Bulgaria

**Keywords:** tautomerism, proton transfer, excited-state, spectroscopy

## Abstract

In this short review, we attempt to unfold various aspects of excited-state intramolecular proton transfer (ESIPT) from the studies that are available up to date. Since Weller’s discovery of ESIPT in salicylic acid (SA) and its derivative methyl salicylate (MS), numerous studies have emerged on the topic and it has become an attractive field of research because of its manifold applications. Here, we discuss some critical aspects of ESIPT and tautomerization from the mechanistic viewpoint. We address excitation wavelength dependence, anti-Kasha ESIPT, fast and slow ESIPT, reversibility and irreversibility of ESIPT, hydrogen bonding and geometrical factors, excited-state double proton transfer (ESDPT), concerted and stepwise ESDPT.

## 1. Introduction

Excited-State Proton Transfer (ESPT) is an important reaction that controls the functioning of various biological systems [1,2,3,4,5,6,7,8,9]. Probe molecules in various biological systems, based on this mechanism, have also been suggested recently [10,11]. Moreover, ESIPT systems have found applicability in sensors for humidity [12,13], luminescent solar collectors [14], proton transfer lasers [15,16,17,18,19], photo stabilizers [20], devices based on thermally activated delayed fluorescence [21,22], white light generation [23,24,25], organic light-emitting diodes (WOLED) [26] as well as suitability in sensing of anion and cations [27,28,29,30], photochromic switching [31,32] and even understanding of fading of colorants in art [33].

**Scheme 1:**
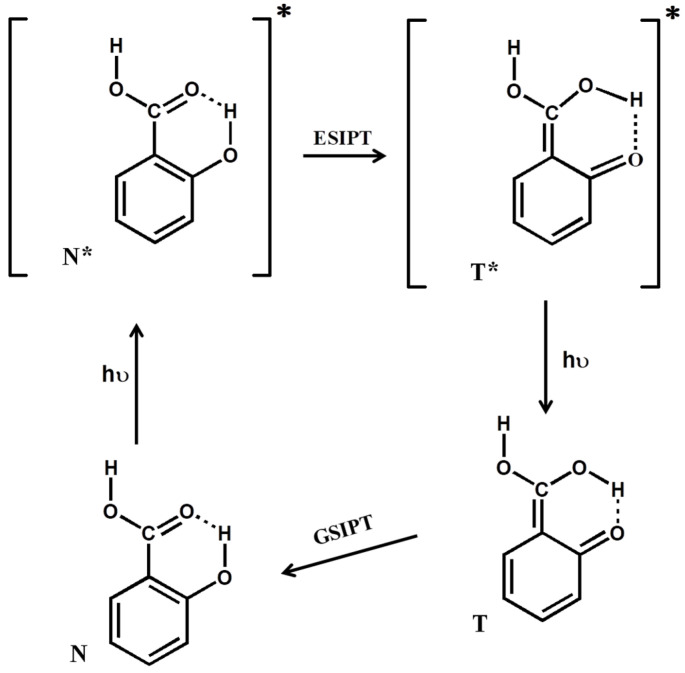
Representative photocycle of excited-state intramolecular proton transfer (ESIPT) in salicylic acid (SA).

As a general definition, molecules, which have both proton (hydrogen) donating/accepting groups, can undergo excited-state intramolecular proton transfer (ESIPT) due to increased acidity/basicity. In salicylic acid (SA), for example, the hydroxyl hydrogen becomes more electropositive whereas the carboxylic oxygen attains more electronegative character in the excited-state. This results in proton translocation from the hydroxyl group to the carboxylic group. A more general schematic representation of ESIPT in SA is given in Scheme 1. Here N and T are the ground state normal and tautomer (obtained as a result of the ESIPT) forms, respectively. Their excited state analogues are denoted as N* and T*. This translocation may be complete resulting in zwitterion [34,35] involving electron transfer and in some cases, it is displaced, resulting in partial or complete hydrogen transfer (ESIHT). A clear distinction between ESIHT and ESIPT is pointed out by Waluk [36]. 

Traditionally, ESIPT is considered as a process where the proton is exchanged through intramolecular hydrogen bonding between the proton donating and accepting groups in the molecule. There are also systems, where the proton donating and accepting sites are not adjacent and hence the proton is transferred over a long distance. In this particular case, the PT can be either solvent or concentration assisted and as a net effect a proton is transferred in the same molecule, termed as pseudo-intramolecular [37]. 

The families of hydroxy carboxylic acids that have been exploited as proton transfer systems are hydroxyl-benzoic acids and esters [34,35,38,39,40,41,42,43,44,45,46,47,48,49,50,51,52,53,54,55,56,57,58,59,60,61,62,63,64,65,66,67,68,69,70], hydroxynaphthoic acids [71,72,73,74,75] and anthranilic acid and its esters [76,77,78,79,80,81]. Besides carboxylic acids, other compounds belonging to hydroxyflavones [82,83,84], quinoline family and anthraquiniones [85,86,87,88,89,90,91,92,93,94,95,96,97,98,99,100], 7-azaindole and derivatives, hydroxyl phenyl benzoxazole(HBO) derivatives [100,101,102,103,104,105,106,107,108,109,110,111,112,113,114,115,116,117,118,119]. Azo dyes and Schiff bases [120,121,122,123,124] were extensively studied and are well documented in the literature. Some other notable systems have also been the subject of interest [124,125,126,127,128,129,130,131]. A summary of some of the most frequently used experimental methods is given in [3].

Experimentally, ESIPT can be traced out by the appearance of large Stokes shifted emission. The ESIPT process can be probed by steady-state as well as time-resolved measurements [124]. As ESIPT occurs in femtosecond scale, in most of the cases its time evolution can be studied by fluorescence up conversion technique [84,125] or from line width information from Shpol’skii matrices [83]. For slower ESIPT, time-correlated single photon counting (TCSPC) can provide information about its dynamics.

The aim of the present review is to assess the main aspects regarding ESIPT dynamics. For this reason, this thematic article is divided into the following sections, where some important developments in the field of ESIPT are discussed:

(1) Fast and slow ESIPT vis a vis irreversible and reversible ESIPT
(2) Effect of substitution, hydrogen bonding and geometrical effects
(3) Excited-state double proton transfer (ESDPT), proton relay, concerted/stepwise proton transfer
(4) Excitation wavelength dependence, anti-Kasha ESIPT, anti-aromaticity
(5) Ground state vis a vis excited-state tautomerisation

## 2. Fast and Slow ESIPT

**Figure 1:**
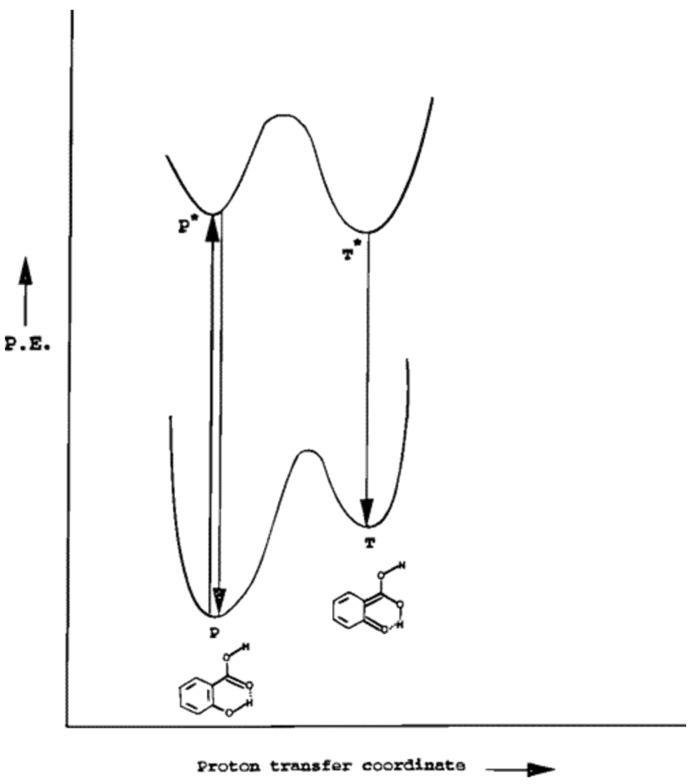
Asymmetric double-well potential for ESIPT in SA and methyl salicylate (MS) proposed by Weller (reproduced by permission from reference [63]). Reprinted with permission from (Maheshwari, S.; Chowdhury, A.; Sathyamurthy, N.; Mishra, H.; Tripathi, H.B.; Panda, M.; Chandrasekhar, J. Ground and Excited-state Intramolecular Proton Transfer in Salicylic Acid: An Ab Initio Electronic Structure Investigation. *J. Phys. Chem. A* **1999**, *103*, 6257–6262). Copyright (1999) American Chemical Society.

In his pioneering work, Weller suggested that there is an asymmetric double-well potential (Figure 1) for the ESIPT in SA and its derivative methyl salicylate (MS) to explain the observed normal (with small Stokes shift) from P* and large Stokes shifted emission resulting from the tautomer T* after ESIPT [34,35]. 

**Figure 2:**
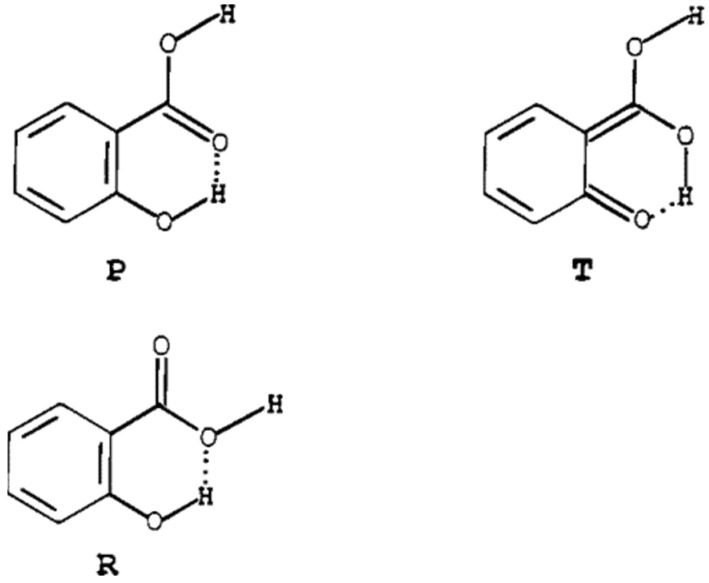
P, R and T forms of SA from reference [63]. Reprinted with permission from (Maheshwari, S.; Chowdhury, A.; Sathyamurthy, N.; Mishra, H.; Tripathi, H.B.; Panda, M.; Chandrasekhar, J. Ground and Excited-state Intramolecular Proton Transfer in Salicylic Acid: An Ab Initio Electronic Structure Investigation. *J. Phys. Chem. A* **1999**, *103*, 6257–6262). Copyright (1999) American Chemical Society.

However, later, careful excitation spectra as well as time-resolved studies revealed that the concept of double-well potential was not correct in these cases, and in fact, normal and tautomer emissions result from two different conformers—one with a proper intramolecular hydrogen bond (IHB) to undergo ESIPT (P form) resulting in T form and the other lacking this strong IHB and giving rise to normal emission (R form) [63] as shown in Figure 2. The emission from the T form has a large Stokes shift whereas normal emission from the R form has a rather small Stokes shift. Moreover, the excitation spectrum corresponding to the emission from R form is red-shifted as compared to the excitation spectrum corresponding to T form [50,53]. Time-resolved studies showed that ESIPT is very fast [40] and occurs in the fs scale, which is now considered as a typical feature of this phenomenon [40,83,84].

**Figure 3:**
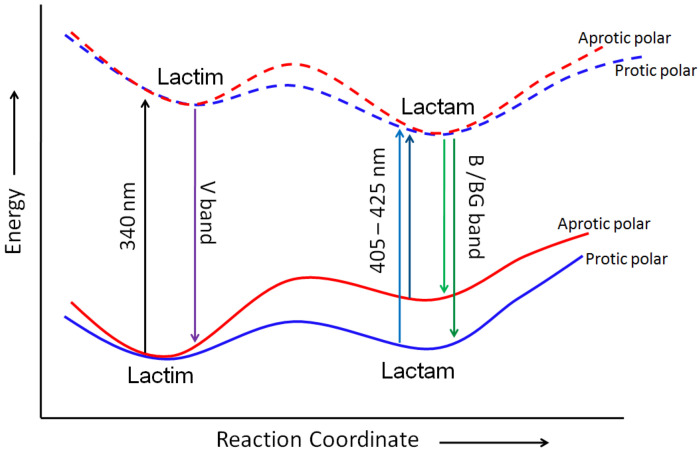
Illustration of solvent-dependent ESIPT in 3-HQ ( the potential barrier is increased in case of aprotic polar solvent as compared protic polar) from ref [89].

However, some studies suggested a slow ESIPT occurring in certain molecules. Alarcos et al. [105] reported that due to intramolecular charge transfer (ICT), the proton transfer is slowed down in 6-amino-2-(2’-hydroxyphenyl)benzoxazole (6A-HBO) and its methylated derivatives and occurs in picosecond time scale. In the case of methanol, it has been found to be assisted by solvent molecules and occurs through tunneling as a large kinetic isotope effect (KIE) of about 13 is observed. Joshi et al. [89] found that ESIPT is rather slow in 3-hydroxyisoquinoline (3-HQ) and can be observed in nanosecond scale depending on solvent polarity. An illustration of this is shown in Figure 3. 

Slow proton transfer dynamics in the nanosecond scale was also reported for 1-(2-hydroxy-5-chloro-phenyl)-3,5-dioxo-1*H*-imidazo-[3,4-*b*]isoindol(ADCL) by Ray et al. [95]. Khimich et al. [93] have shown that 2-amino-3-(2’-benzoxazolyl)-quinoline (ABO) upon protonation exhibits dual fluorescence resulting from initial tautomer and ESIPT product. Moreover, the cation has a significant potential barrier which decreases with an increase in basicity according to the theoretical calculations.

**Figure 4:**
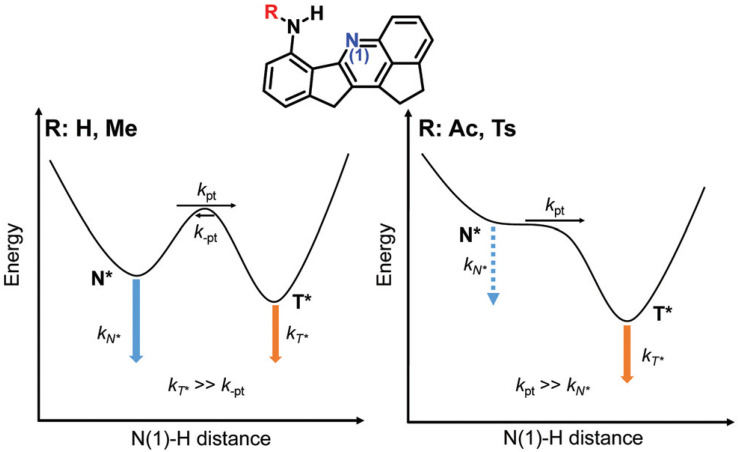
Effect of substitution on ESIPT in 2,11-dihydro-1*H*-cyclopenta[*de*]indeno[1,2-*b*]quinoline (CPIQ) from ref. [92]. Reprinted with permission from (Liu, Z.-Y.; Hu, J.-W.; Huang, T.-H.; Chen, K.-Y.; Chou, P.-T. Excited-State Intramolecular Proton Transfer in the Kinetic-Control Regime. *Phys. Chem. Chem. Phys.* **2020**, *22*, 22271–22278). Published by the PCCP Owner Societies.

In 3-hydroxychromene (3-HC), intermolecular interactions have been found to slow down the ESIPT reaction considerably [94]. ESIPT process in 3-hydroxy-2-methylbenzo[*g*]quinolin-4(1*H*)-one (MBQ) was studied by Zamotaiev et al. [86] who found that reversible ESIPT with a rather long time scale (ns) takes place. Liu et al. [92] investigated ESIPT in a series of molecules bearing a 2,11-dihydro-1*H*-cyclopenta[*de*]indeno[1,2-*b*]quinoline (CPIQ) chromophore and found that ESIPT is irreversible with a significant barrier (kinetic-control regime). Moreover, ESIPT can be tuned by the substituent. The representative reaction is demonstrated in Figure 4.

**Figure 5:**
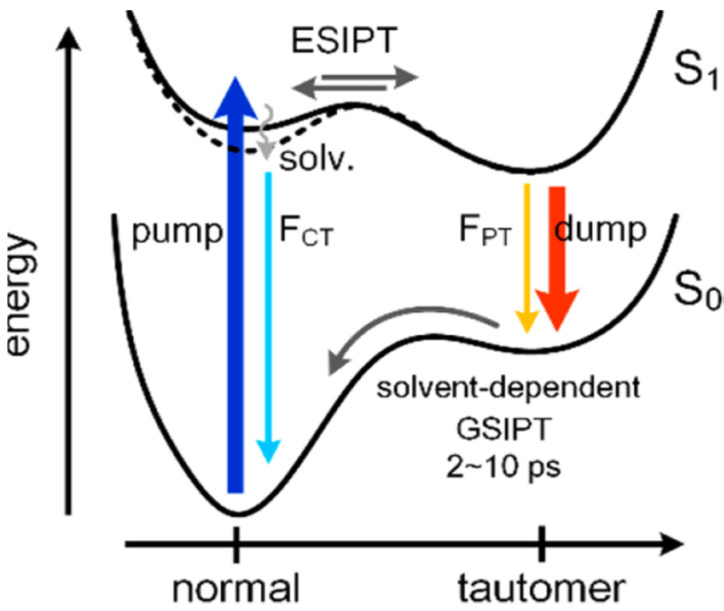
Solvent polarity effect on ESIPT and ground state intramolecular proton transfer (GSIPT) in 4′-*N,N*-diethylamino-3-hydroxyflavone (DEAHF) from ref [90]. Reprinted with permission from (Kuang, Z.; Guo, Q.; Wang, X.; Song, H.; Maroncelli, M.; Xia, A. Ultrafast Ground-State Intramolecular Proton Transfer in Diethylaminohydroxyflavone Resolved with Pump–Dump–Probe Spectroscopy. *J. Phys. Chem. Lett.* **2018**, *9*, 4174–4181). Copyright (2018) American Chemical Society.

Ni et al. [97] modelled the effect of substitution on ESIPT behaviour in 4-(2-hydroxybenzylidene)-1,2-dimethyl-1*H*-imidazol-5(4*H*)-one (o-LHBDI) derivatives by DFT and TD-DFT methods. They found that the ESIPT barrier decreases when the substituent has a stronger electron-withdrawing ability or weaker electron-donating ability. Zhang et al. [96] investigated ESIPT on “naked” diazaborepins and found that ESIPT is prohibited with an increase in solvent polarity. Solvent-dependent ESIPT and ground state intramolecular proton transfer (GSIPT) has been reported by Kuang et al. in 4′-*N,N*-diethylamino-3-hydroxyflavone (DEAHF) [90]. They used femtosecond pump–dump probe spectroscopy to prepare short-lived grounds state tautomers and monitor the GSIPT process. Moreover, GSIPT has been found to be an irreversible two-state process. A representative diagram showing ESIPT and GSIPT is shown in Figure 5.

## 3. Hydrogen Bonding, Effect of Substitution and Crystal Structure

ESIPT reaction has been found to depend on external hydrogen bonding with the molecule. In SA, external hydrogen bonding with diethyl ether (Et_2_O) breaks the existing dimers and only monomeric emission from tautomer has been observed with enhanced intensity [51]. The effect of substitution on SA was studied extensively, indicating the ESIPT dependence. Lahmani and Zehnacker-Rentien [54] studied the effect of methyl and methoxy substitution at the para position of the hydroxyl group in SA. They found that methoxy salicylic acid (5-MeOSA) shows only normal emission (~400 nm) but dual emission including emission from tautomer (500 nm) is observed in the presence of hydrogen bonding diethyl ether and suggested that hydrogen bonding promotes ESIPT reaction. Smoluch et al. extended the study with time-resolved measurements for 5-MeOSA and suggested a geometric change on excitation. Table 1 shows the decay data of 5-MeOSA. It can be seen that there is a rising component in the decay profile of tautomer emission at 298 K which, however, is not evident at 160 K. Instead a mono-exponential decay is observed. The presence of rising component at 298 K has been attributed to excited-state equilibrium between normal and tautomer emissions which apparently is not present at 160 K. These observations indicate that besides hydrogen bonding, molecular geometry also plays an important role in ESIPT. Fluorescence properties of the salicylic anion have also been found to be modulated considerably depending on electron donor/acceptor substituents [66]. In polymer matrix (PMMA), SA has been found to exhibit only B emission arising from tautomer (T) after ESIPT. The absence of dimer is ascribed to hydrogen bonding with the polymer [51]. However, it shows a bi-exponential decay throughout the emission profile indicating the effect of heterogeneity in polymeric media. Again these observations point to the fact that both hydrogen bond and its geometry play an important role in ESIPT.

Parada et al. [98] reported the influence of hydrogen bond geometry on the dynamics of ESIPT in phenol-quinoline compounds. They found that long donor-acceptor distance increases the PT barrier whereas a large dihedral angle (angle between the planes having proton donor-acceptor subunits) results in a deactivation channel after ESIPT. 

Hydrogen bonding wires/bridge formation has been reported to be a prerequisite for ESIPT in quinolone derivatives, where proton donor and acceptor groups are not adjacent. 6-hydroxyquinoline (6-HQ) and 7-hydroxyquinoline (7-HQ) have been found to undergo ESIPT in water, methanol: water and acetic acid through hydrogen bridge formation [99,101,102,103,104]. Although the proton is transferred through intermolecular hydrogen bonding(s) the process as a net effect can be considered as intramolecular. Therefore, these can be considered as cases of solvent-assisted pseudo intramolecular excited-state proton transfer.

Hristova et al. [87,88] studied ESIPT in 10-hydroxybenzo(h)quinolone (HBQ) and structurally modified compounds. They found that keto tautomer is observed when electron acceptor substituents are present at position 7 of the HBQ backbone. Theoretical modelling suggested that the substitution results in a transition from a single well to double minimum potential. They have attributed it to the charge transfer character imparted by the substituent.

**Figure 6:**
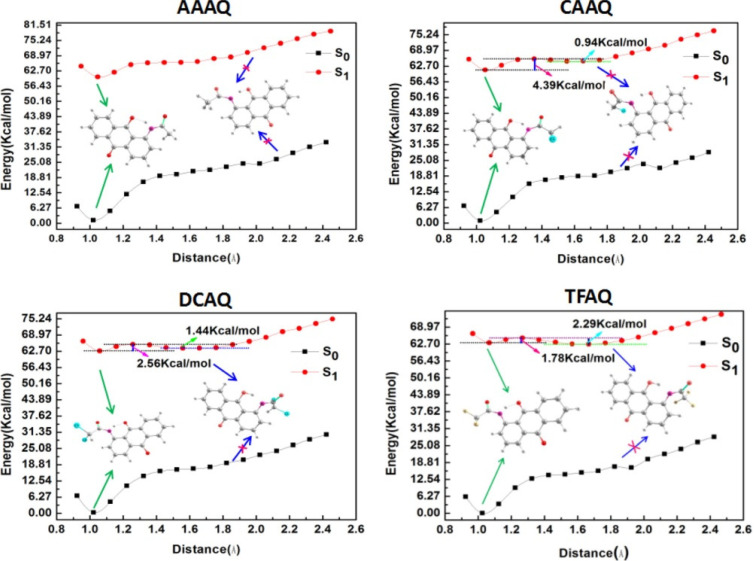
Theoretically estimated potential energy surfaces for AAQ’s (reproduced from reference [126]. Reprinted with permission from (Ma, Y.; Yang, Y.; Lan, R.; Li, Y. Effect of Different Substituted Groups on Excited-State Intramolecular Proton Transfer of 1-(Acylamino)-Anthraquinons. *J. Phys. Chem. C* **2017**, *121*, 14779–14786). Copyright (2017) American Chemical Society.

Effect of different substituted groups on ESIPT in 1-(aceylamino)-anthraquinones (AAQ’s) was studied theoretically by Ma et al. [126]. They reported that hydrogen bond strengthening was followed by ESIPT depending on the potential energy barrier of a particular derivative. Theoretically computed potential energy surfaces (PES) are shown in Figure 6. It can be seen that the potential barrier in the S_1_ state is significantly affected by substitution.

**Figure 7:**
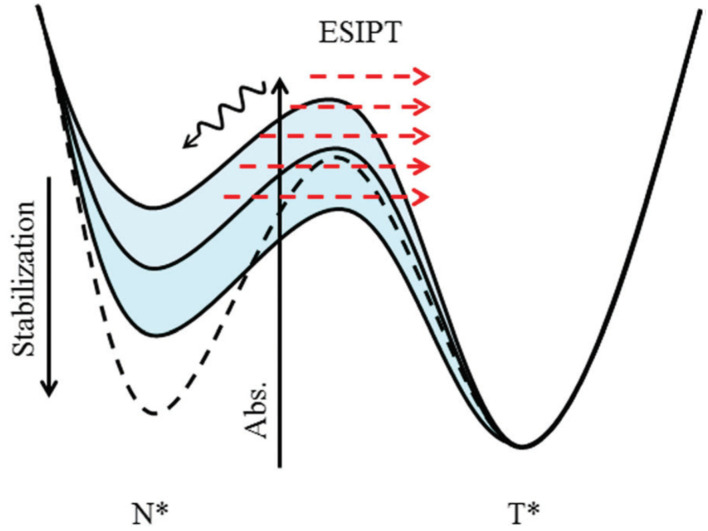
Modification of potential energy surface (PES) by crystalline phases of acetonitrile from reference [85]. Solid and dotted lines show potential energy curves for low and high-temperature crystalline phases respectively. Reprinted with permission from (Furukawa, K.; Yamamoto, N.; Hino, K.; Sekiya, H. Excited-State Intramolecular Proton Transfer and Conformational Relaxation in 4′-N,N-Dimethylamino-3-Hydroxyflavone Doped in Acetonitrile Crystals. *Phys. Chem. Chem. Phys.* **2016**, *18*, 28564–28575). Published by the PCCP Owner Societies.

The effect of intermolecular interactions on excited-state intramolecular proton transfer (ESIPT) in 4’-*N,N*-dimethylamino-3-hydroxyflavone (DMHF) doped in acetonitrile crystals was studied by Furukawa et al. [85]. They found prominent differences in two different crystalline phases which are explained by modulation of the potential energy surface (Figure 7). In a recent work, the structure–property relationship on the ESIPT mechanism from the viewpoint of the design of efficient solid-state emitters is elucidated. They have demonstrated that besides restricting the nonradiative pathways, an efficient ESIPT is also essential for efficient emitters [106].

## 4. Excited-State Double Proton Transfer (ESDPT), Proton Relay, Concerted/Stepwise Proton Transfer

**Figure 8:**
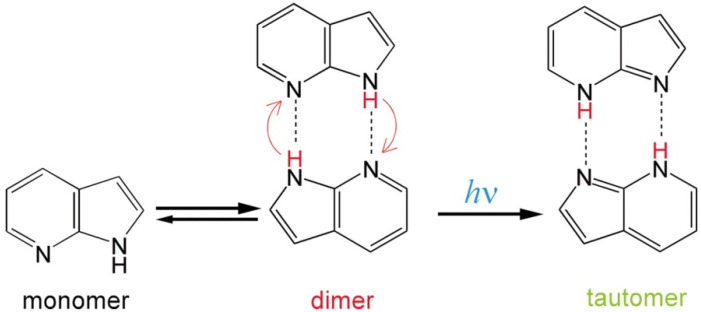
7-AI monomer, dimer and tautomer forms from reference [113]. Reprinted with permission from (Takeuchi, S.; Tahara, T. The Answer to Concerted versus Step-Wise Controversy for the Double Proton Transfer Mechanism of 7-Azaindole Dimer in Solution. *Proceedings of the National Academy of Sciences* **2007**, *104*, 5285–5290). Copyright (2007) National Academy of Sciences, U.S.A.

**Figure 9:**
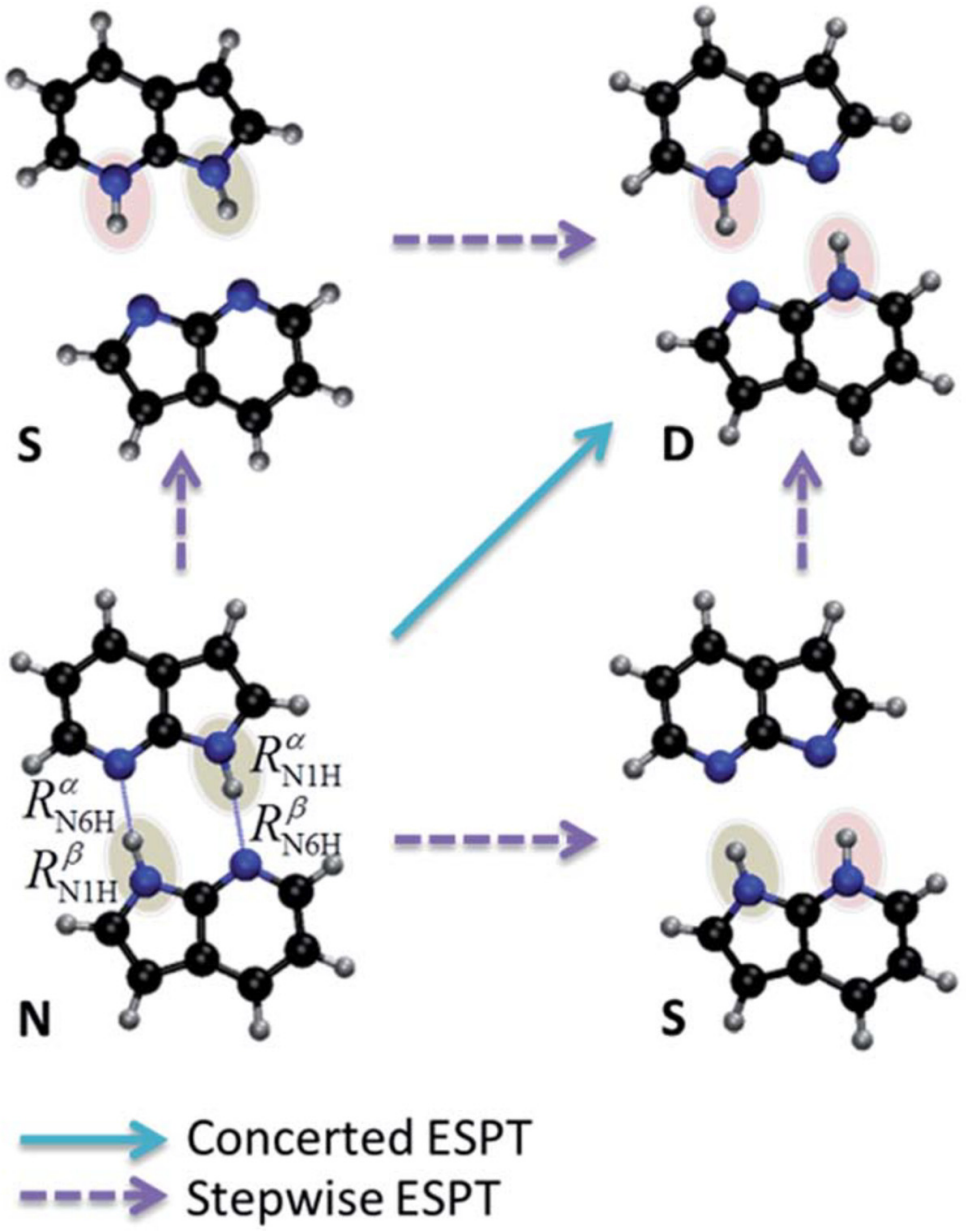
Demonstrating stepwise and concerted ESDPT in 7-AI (adapted from reference [114]). Here N is the dimer, S is the intermediate molecule resulting from single proton transfer and D is the tautomer resulting from ESDPT. Reprinted with permission from (Crespo-Otero, R.; Kungwan, N.; Barbatti, M. Stepwise Double Excited-State Proton Transfer Is Not Possible in 7-Azaindole Dimer. *Chem. Sci.* **2015**, *6*, 5762–5767). Published by The Royal Society of Chemistry.

ESDPT is also considered to represent typical cases of concentration-assisted ESIPT occurring in dimers. ESDPT can be traced back to the work of Taylor et al. [107] in 7-azaindole (7-AI) which they attributed to cooperative biprotonic reversible proton transfer. Since then, several studies were done to elucidate the presence of ESDPT [108,109,110,111,112,113,114,127,128,129,130,131]. Figure 8 shows 7-AI monomer, dimer and tautomer formed by ESDPT. The proton transfer time was found to be rather short (ps time scale) [109]. The ESDPT process can occur either in a stepwise manner or through a concerted mechanism (illustrated in Figure 9). By femtosecond time-resolved measurements, Douhal et al. [110] suggested that in 7-AI, ESDPT takes place in a stepwise manner. The mechanism remained controversial [111,112] until new experimental as well theoretical findings indicated that it occurs in a concerted fashion. From time-resolved measurements, Takeuchi and Tahara [113] concluded that ESDPT in 7-AI dimers takes place in a concerted manner in 1.1 ps time. By using a proper topological description of the 7-AI dimer in the gas phase, Crespo-Otero et al. [114] suggested that stepwise ESDPT is not favorable due to kinetic and thermodynamical reasons.

A detailed account of ESDPT has also been reported recently showing that some systems appear to favour stepwise ESDPT mechanism [115]. For ESDPT in 2-aminopyrene/acetic acid (2-AP) system, three types of ESDPT are suggested. Besides concerted, stepwise 1 and stepwise 2 ESDPT (depending on the sequence of proton transfers) are pointed out. From their calculations, they found that the first proton transfer is barrierless (stepwise 1) whereas a high barrier exists for concerted and stepwise 2 processes. 

In another work, the ESIPT mechanism in 2-(2-hydroxyphenyl)benzoxazole (HBO), bis-2,5-(2-benzoxazolyl)-hydroquinone (BBHQ) and 2,5-bis(5’-tert-butyl-benzoxazol-2’-yl)hydroquinone (DHBO) were investigated using time-dependent density functional theory (TDDFT). The possibility of simultaneous ESDPT or successive proton transfers besides single proton transfer is reported [128].

The ESDPT mechanism in 2,2’-Bipyridyl-3,3’-diol (BP(OH)2) has been examined theoretically by Zhao et al. [129] in different polar aprotic solvents and found that solvent can regulate and control the ESDPT process. Plasser et al. [130] studied the ESDPT process in BP(OH)_2_ by second-order approximate coupled-cluster (RI-CC2) and time-dependent density functional theory (TDDFT) and suggested that concerted process in this molecule is highly unlikely as it proceeds along a ridge in the S_1_ excited-state and advocated for stepwise mechanism.

The other notable compound is SA which also forms cyclic dimers in the crystalline state. Pant et al. [46] suggested that ESDPT may be responsible for the tautomer emission. Bisht and co-workers in a supersonic jet study also reported the possibility of double proton transfer in excited-state [50]. Later studies also confirmed the tautomer emission in dimers, but could not find the evidence of ESDPT in SA systems [47,48,61]. Young et al. suggested a conical intersection (CI) in the case of 5-fluorosalicylic acid dimer [48]. In view of these studies to further explore the ESDPT mechanism from both theoretical as well as experimental points of view will be interesting.

Other interesting examples of tautomerization are porphycenes which exhibit ground as well as excited-state double proton transfer [131]. This is manifested in depolarized emission, viscosity-dependent radiationless deactivation and vibrational mode-specific tunneling splitting.

## 5. Excitation Wavelength Dependence

Excitation wavelength dependence on the excited-state proton transfer was first reported by Hetherington et al. in 7-azaindole [98,109]. They found that excited-state double proton transfer occurs in less than 5 ps at room temperature, but exhibits excitation wavelength dependence at 77 K which could be explained on the basis of a double-well potential. The investigation suggests that direct (from the initially excited state) and indirect (from the relaxed excited state) emissions take place which explains the presence of slow component at 77 K. Pant et al. [46] investigated the excitation wavelength dependence in SA solid. Two fluorescence bands U (~370 nm) and B (~450 nm) are observed. They attributed U emission to the molecules to the dimer molecules which do not undergo ESIPT whereas the B emission which has a large Stokes shift arising from ESIPT. They found that at room temperature as well as for shorter excitation wavelengths at 77 K, the ratio of U to B intensities remains unchanged. However, at 77 K and for longer wavelength excitation, the B emission changes drastically. Further, the decay for both normal and tautomeric emissions is exponential with identical decay times at higher temperatures as well as for shorter wavelengths of excitation. This indicates that there exists a fast excited-state equilibrium between normal and proton transferred forms. The presence of ground state tautomers and fast inter-conversion was suggested as a possible reason. In a later study, Denisov et al. [47] also investigated temperature and excitation wavelength dependence in SA and dihydroxy-benzoic acid (DHBA) and possibilities for explaining this behaviour were pointed out. They pointed out that for a self-consistent interpretation of observations, the excited state of the dimer may have double-well potential with a rather high barrier. This may arise from the organization of the dimer geometry upon excitation. However, to address the temperature and excitation wavelength dependence a comprehensive theoretical study is still lacking and needs to be explored further. The role of anti-aromaticity on ESIPT has also been elucidated in a recent work [49]. It has been suggested that SA is aromatic in S_0_ and S_2_ states but the ESIPT can occur only from S_1_ state, which is antiaromatic. This should be an interesting aspect if this holds for SA dimers also. 

**Figure 10:**
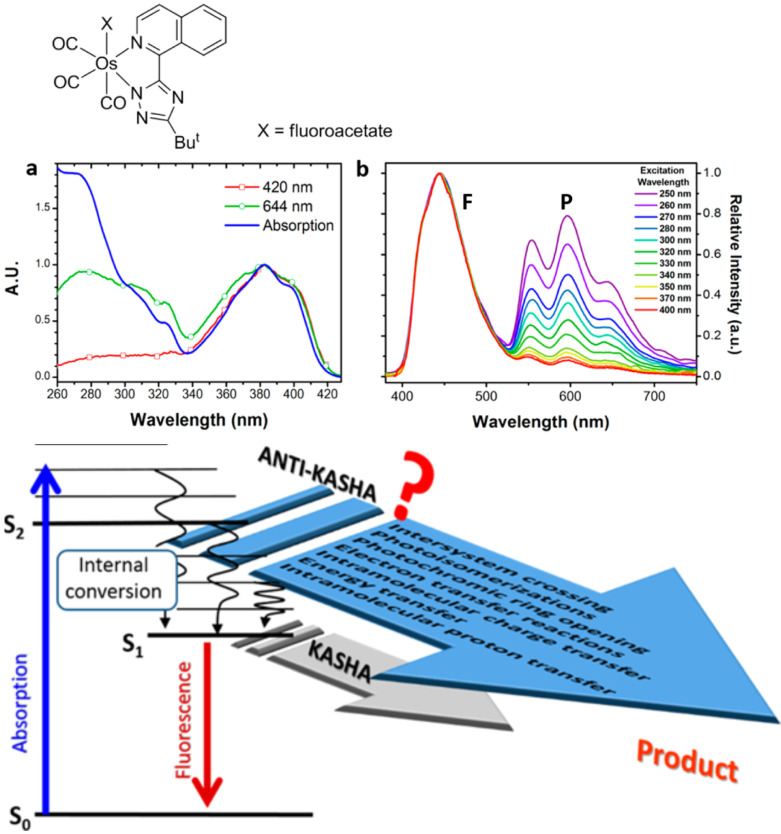
Demonstrating anti-Kasha ESIPT (enhanced emission at shorter excitation wavelengths) from reference [116]: (**a**) absorption and excitation spectra; (**b**) emissions spectra. Reprinted with permission from (Demchenko, A.P.; Tomin, V.I.; Chou, P.-T. Breaking the Kasha Rule for More Efficient Photochemistry. *Chem. Rev.* **2017**, *117*, 13353–13381). Copyright (2107) American Chemical Society.

Recently renewed interest in the excitation wavelength dependence of the ESIPT emerged in the literature [116,117,118]. Excitation wavelength dependence on the ESIPT emission was observed and reported as anti-Kasha ESIPT (Figure 10). As Kasha’s rule states that emission originates from the lowest excited singlet state, the ESIPT emission is observed from the lowest excited state of tautomer after undergoing ESIPT from normal molecule to the S_1_ state of tautomer.

**Figure 11:**
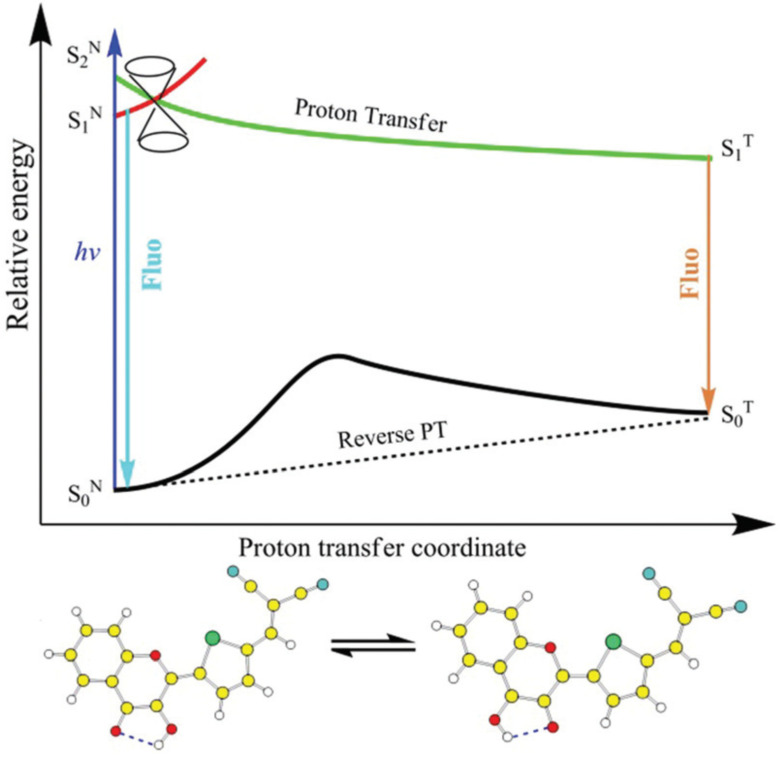
Theoretical calculations demonstrating ESIPT from higher excited states from reference [118]. Reprinted with permission from (Yuan, H.; Guo, X.; Zhang, J. *Ab Initio* Insights into the Mechanism of Excited-State Intramolecular Proton Transfer Triggered by the Second Excited Singlet State of a Fluorescent Dye: An anti-Kasha Behavior. *Mater. Chem. Front.* **2019**, *3*, 1225–1230. Published by The Royal Society of Chemistry.

Moreover, some theoretical attempts have also been made to explain the excitation wavelength dependence [118]. They have attributed the increased quantum yield from the tautomer emission as a result of anti-Kasha ESIPT. It appears, as shown in Figure 11, to be a potential energy surface crossing between the S_2_ state of the normal molecule and S_1_ state of the tautomer. It is clearly evident that from higher excited-states ESIPT is enhanced, however, terming it as anti-Kasha is debated [119]. 

## 6. Ground State vis a vis Excited-State Tautomerization

Certain categories of molecules, such as azo dyes and Schiff bases, have also been found to form tautomers in both ground and excited states [120,121,122,123,124] 

**Scheme 2:**
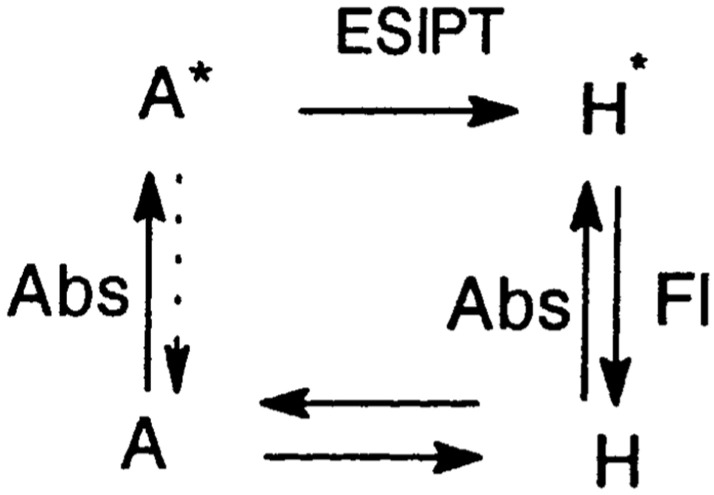
Representative scheme for ground and excited-state tautomerization (A and H represent azo and hydrazone tautomers respectively: A* and H* are corresponding excited-state counterparts) [121].

As these systems involve both ground and excited tautomerisation [132] (as sketched by Scheme 2), the efficiency of tautomerization in the excited state is an important parameter. In these works, the authors estimated the efficiency of tautomerization from the excitation spectra. Accordingly, in the case of ESIPT, the excitation spectrum will have a contribution from both enol-like (A) and keto-like (H) forms with emission coming only from the H tautomer. In this case, we can express the efficiency (η) by [121]:

(1) $$\eta =\frac{I_{\mathrm{exc}}\left(A\right).A_{H}}{I_{\mathrm{exc}}\left(H\right).A_{A}}\times 100$$

where A_A_ and A_H_ are the measured absorbances at the maxima of A and H respectively and I_exc_(A) and I_exc_(H) are the excitation intensities at the same wavelengths. This approach has been adopted to estimate excited-state tautomerization in other systems also [122,123,124].

## 7. Conclusions and Outlook

In this short review, we have briefly summarized some valuable contributions encompassing critical issues associated with ESIPT.The covered issues are slow and fast ESIPT, effect of hydrogen bonding, substitution and crystal structure (in crystalline phase), ESDPT and its mechanism, excitation wavelength dependence on ESIPT, the role of conical interactions in ESIPT and ground and excited-state tautomerism. We believe the review will provide a platform for furthering efforts in ESIPT from both mechanistic and application points of view. 

## Figures and Tables

**Table 1:** Fluorescence decay parameters of 5-MeOSA-Et_2_O complex. (τ_1_ and τ_2_ are decay rising components and α_1_ and α_2_ are corresponding amplitudes).

Temperature K	Emission Wavelength nm	τ_1_ ns	τ_2_ ns	α_1_	α_2_	χ^2^
298 *	400	0.4	2.32	51.52	48.58	1.04
298 *	500	0.43	2.42	−5.27	14.41	1.09
160 **	400	2.51	11.54	31.55	68.45	1.0
160 **	500	-	11.50	-	-	1.05

From: * decay data are shown in the corresponding channel from reference [55]; ** unpublished decay data.

## Data Availability

Data sharing is not applicable to this article.
